# Containment efficiency and control strategies for the corona pandemic costs

**DOI:** 10.1038/s41598-021-86072-x

**Published:** 2021-03-25

**Authors:** Claudius Gros, Roser Valenti, Lukas Schneider, Kilian Valenti, Daniel Gros

**Affiliations:** 1 Institute of Theoretical Physics, Goethe University, 60438 Frankfurt, Germany; 2 Vivantes Klinikum Spandau, 13585 Berlin, Germany; 3 Department of Economics, University of California, Berkeley, USA; 4 CEPS (Centre for European Policy Studies), 1000 Brussels, Belgium

**Keywords:** Diseases, Mathematics and computing, Physics

## Abstract

The rapid spread of the Coronavirus (COVID-19) confronts policy makers with the problem of measuring the effectiveness of containment strategies, balancing public health considerations with the economic costs of social distancing measures. We introduce a modified epidemic model that we name the controlled-SIR model, in which the disease reproduction rate evolves dynamically in response to political and societal reactions. An analytic solution is presented. The model reproduces official COVID-19 cases counts of a large number of regions and countries that surpassed the first peak of the outbreak. A single unbiased feedback parameter is extracted from field data and used to formulate an index that measures the efficiency of containment strategies (the CEI index). CEI values for a range of countries are given. For two variants of the controlled-SIR model, detailed estimates of the total medical and socio-economic costs are evaluated over the entire course of the epidemic. Costs comprise medical care cost, the economic cost of social distancing, as well as the economic value of lives saved. Under plausible parameters, strict measures fare better than a hands-off policy. Strategies based on current case numbers lead to substantially higher total costs than strategies based on the overall history of the epidemic.

## Introduction

In March 2020 the World Health Organization (WHO) declared the Coronavirus (COVID-19) outbreak a pandemic^1^. In response to the growth of infections and in particular to the exponential increase in deaths^2^, a large number of countries were put under lockdown, leading to an unprecedente recession^3^ which could potentially have longer term costs^4^. In this situation it is paramount to provide scientists, the general public and policy makers with reliable estimates of both the efficiency of containment measures (*e.g.* social distancing and non-pharmaceutical health interventions), and the overall costs resulting from alternative strategies.

**Figure 1:**
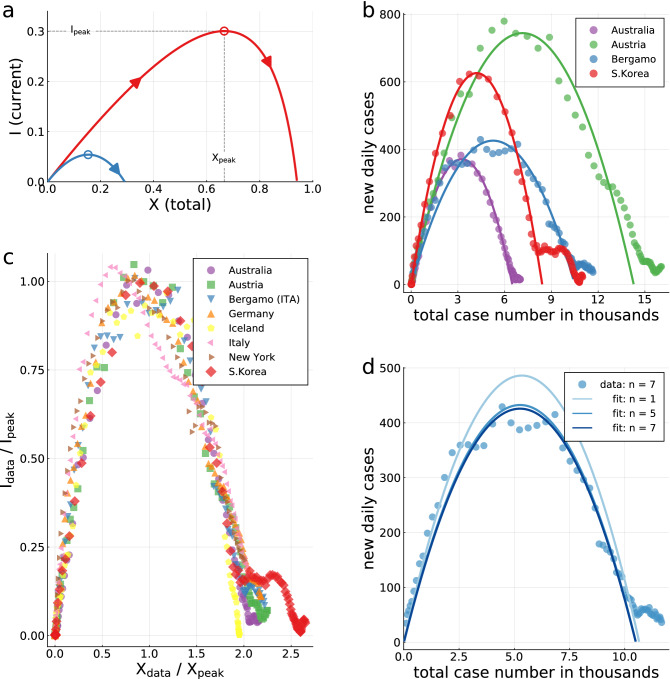
XI representation of COVID-19 outbreaks. (**a**) Model illustration. The closed phase-space expression $I=I(X)$ of actual infected cases *I* as a function of total infected cases *X*, as given by Eq. (3), is shown for two cases: $\alpha _X=0$ (no control, red line) and $\alpha _X=10$ (long-term control, blue line) for an intrinsic reproduction factor of $g_0=3$. The number of infections is maximal at $I_{\mathrm{peak}}$ (open circle), after starting at $X=I=0$, with the epidemic ending when the number of actual cases drops again to zero. At this point the number of infected reaches $X_{\mathrm{tot}}$. The peak $X_{\mathrm{peak}}=2/3$ of the uncontrolled case, $\alpha _X=0$, is sometimes called the ‘herd immunity’ point. The final fraction of infected is $X_{\mathrm{tot}}=0.94$. (**b**) Model validation for a choice of four countries/regions. The model (lines) fits the seven-day centered averages of COVID-19 case counts well. For South Korea data till March 10 (2020) has been used for the XI-fit, at which point a transition from overall control to the tracking of individuals is observable. (**c**) Data collapse for ten countries/regions. Rescaling with the peak values $X_{\mathrm{peak}}$ and $I_{\mathrm{peak}}$, obtained from the XI fit, maps COVID-19 case counts approximately onto a universal inverted parabola. (**d**) Robustness test. The often strong daily fluctuations are smoothed by *n*-day centered averages. Shown are the Bergamo data (dots, $n=7$) and XI-fits to $n=1$ (no average), $n=5$ and $n=7$. Convergence of the XI-representation is observed.

The societal and political response to a major outbreak like COVID-19 is highly dynamic, changing often rapidly with increasing case numbers. We propose to model the feedback of spontaneous societal and political reactions by a standard epidemic model that is modified in one key point: the reproduction rate of the virus is not constant, but evolves over time alongside with the disease in a way that leads to a ‘flattening of the curve’^5^. The basis of our investigation is the SIR (Susceptible, Infected, Recovered) model, which describes the evolution of a contagious disease for which immunity is substantially longer than the time-scale of the outbreak^6^. A negative feedback-loop between the severity of the outbreak and the reproduction factor *g* is then introduced. As a function of the control strength $\alpha _X$, which unites the effect of individual, social and political reactions to disease spreading, the difference between an uncontrolled epidemic ($\alpha _X=0$) and a strongly contained outbreak (large $\alpha _X$) is described, as illustrated in Fig. 1a. The model, which we name controlled-SIR model due to the presence of the control parameter $\alpha _X$, is validated using publicly available COVID-19 case counts from a large range of countries and regions. We provide evidence for data collapse when case counts of distinct outbreaks are rescaled with regard to their peak values. A comprehensive theoretical description based on an analytic solution of the controlled-SIR model is given. One finds substantial differences in the country-specific intrinsic reproduction factor and its doubling time. The controlled-SIR model allows in addition to formulate an unbiased benchmark for the effectiveness of containment measures, the containment efficiency index (CEI).

The controlled-SIR model is thoroughly embedded in epidemiology modeling. Early on, the study of the dynamics of measles epidemics^7^ has shown that human behavior needs to be taken into account^8,9^. In this regard, a range of extensions to the underlying SIR model have been proposed, such as including the effect of vaccination, contact-frequency reduction and quarantine^10^, human mobility^11^, self-isolation^12^, the effects of social and geographic networks^13^, the effects of awareness diffusion and epidemic propagation^14,15^, and the influence of explicit feedback loops^16^. For an in-depth description, epidemiology models need to cover a range of aspects^17^, as the distinction between symptomatic and asymptomatic cases^18^, which prevents in general the possibility of an explicit analytic handling. In the present work we pursue the alternative approach of retaining a minimal set of parameters, such that the resulting epidemiological model allows for an analytical description of the pandemic and its socio-economical aspects.

Political efforts to contain the pandemic, as social-distancing measures and non-pharmaceutical health interventions, are included in the controlled-SIR model as a dampening feedback mechanism. The controlled-SIR model is therefore suitable to estimate the overall economic and health-related costs associated with distinct containment strategies, in particular when accumulated over the entire course of an epidemic outbreak. This approach, which is followed here, extends classical studies of the economic aspects of controlling contagious diseases. A central question regards in this context the weighting of the economic costs of containment against the cost of treatment, and the loss of life^19,20^. For the value of life, statistical approaches attribute suitably estimated monetary values to an avoided premature death^21–23^. The resulting framework has been applied to the corona pandemic in several recent contributions in which the evolution of the epidemic has been taken in general as exogenous^24^, relying on estimates for the infection^25^ and case fatality rates^26,27^. Further studies have discussed the relative effectiveness of control measures^25,28–31^, and the possible future course of the disease^32,33^.

## Results

### Controlled-SIR model

**Figure 2:**
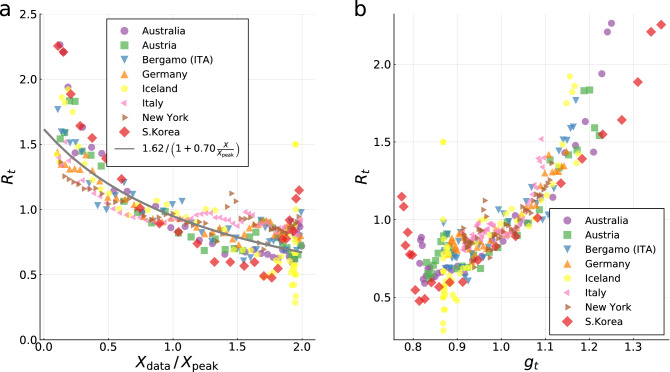
Validation of controlling feedback loop. The fraction of newly infected at time *t* and at $t-4$ is used to estimate the time dependent reproduction factor $R_t = \overline{I}_t / \overline{I}_{t-4}$, when assuming a serial interval of four days (compare^37^). Note that a seven-day centred moving average $\overline{I}_t = \sum _{s=t - 3}^{t+3} I_s$ is utilised. (**a**) $R_t$ as a function of the relative cumulative number $X / X_{\mathrm{peak}}$ of cases. A fit to the same functional form as in Eq. (2) is given (grey line). (**b**) correlation of $R_t$ and $g_t$. The estimated reproduction factor $R_t$ is compared to the effective reproduction factor $g_t$ as defined in Eq. (2). In (**a**) and (**b**) only data between $0.1 \le X / X_{\mathrm{peak}} \le 2$ is shown, with the lower bound discarding the strong fluctuations in the early stages of the pandemic. The upper bound is used to define the termination of the first wave.

In the following we introduce the model. At a given time *t* we denote with $S=S(t)$ the fraction of susceptible (non-infected) individuals and $I=I(t)$ the fraction of the population that is currently ill (active cases). Infected individuals can either recover or die as a consequence of the infection, here we subsume both outcomes under $R=R(t)$, which denotes hence the fraction of recovered or deceased individuals. Normalization demands $S+I+R=1$ at all times. The continuous-time SIR model^34^

1 $$\begin{aligned} \tau \dot{S} = -gSI, \quad \quad \tau \dot{I} = (gS-1)I, \quad \quad \tau \dot{R} = I \end{aligned}$$

describes an isolated epidemic outbreak characterized by a timescale $\tau$ and a dimensionless reproduction factor *g*. Social and political reactions reduce the reproduction factor below its intrinsic (medical disease-growth) value, $g_0$. We describe this functionality as

2 $$\begin{aligned} g = \frac{g_0}{1+\alpha _X X}, \qquad \quad X=1-S\,, \end{aligned}$$

where we generalized standard epidemiological approaches to nonlinear incidence rates^35,36^. The reaction to the epidemic is taken to be triggered by the total fractional case count *X* (i.e. the sum of active, recovered and deceased cases), with $\alpha _X$ encoding the reaction strength. In the Methods section we show how this functionality is validated by COVID-19 data, see also Fig. 2. In this view $\alpha _X$ sums up the effects of an extended number of social processes and political action taking. Further below we will examine in addition strategies for which the response is based on the fraction of actual active cases, *I*. We note that containment due to a reduction in the reservoir of susceptible *S*, is of minor importance, given that COVID-19 infection cases are generally small with respect to the overall population size.

The inverse functionality in Eq. (2) captures the law of diminishing returns, namely that it becomes progressively harder to reduce *g* when increasing social distancing. In this view, small reductions of *g* are comparatively easy, however a suppression by several orders of magnitude requires a near to total lockdown. We denote Eq. (1) together with (2) the controlled-SIR model. Key to our investigation is the observation that one can integrate the controlled-SIR model analytically, as shown in the Methods section, to obtain the phase-space relation

3 $$\begin{aligned} I = \frac{\alpha _X+g_0}{g_0}\,X+ \frac{1+\alpha _X}{g_0}\,\log (1-X)\,. \end{aligned}$$

This relation, which we denote the ‘XI representation’, is manifestly independent of the time scale $\tau$.

The medical peak load $I_{\mathrm{peak}}$ of actual infected cases is reached at a total fractional case count $X= X_{\mathrm{peak}}$, which is given by

4 $$\begin{aligned} gS=1, \qquad \quad X_{\mathrm{peak}} =\frac{g_0-1}{g_0+\alpha _X}\,, \end{aligned}$$

For the case that $\alpha _X=0$ (no control), $X_{\mathrm{peak}}$ reduces to the well-known result $X_{\mathrm{peak}} =(g_0-1)/g_0$.

For finite $\alpha _X$, $I_{\mathrm{peak}}$ is obtained from Eqs. (3) and (4),

5 $$\begin{aligned} I_{\mathrm{peak}} = \frac{g_0-1}{g_0} + \frac{1+\alpha _X}{g_0}\,\log \left( \frac{1+\alpha _X}{g_0+\alpha _X}\right) \,. \end{aligned}$$

For $\alpha _X=0$, $I_{\mathrm{peak}}$ is sometimes called the ’herd immunity point’. The XI representation can be parameterized consequently either by $g_0$ and $\alpha _X$, as in Eq. (3), or indirectly by $X_{\mathrm{peak}}$ and $I_{\mathrm{peak}}$, which are measurable (modulo undercounting). In Fig. 1a an illustration of the XI-representation is given. For $g_0=3$ and $\alpha _X=0$ one has $X_{\mathrm{peak}}=2/3$ and $I_{\mathrm{peak}}\approx 0.3$. The total fraction of infected $X_{\mathrm{tot}}$ is 94%, which implies that only about 6% of the population remains unaffected. Containment policies, $\alpha _X>0$, reduce these values. Fig. 1a and Eq. (5) illustrate a sometimes encountered misconception regarding the meaning of the herd immunity point, which we have labeled simply $I_{\mathrm{peak}}$. The epidemic doesn’t stop at $I_{\mathrm{peak}}$ since infections continue beyond this point, albeit at a declining rate.

### XI representation of COVID-19 outbreaks

In Fig. 1b,c we show for a representative choice of countries, regions and cities that COVID-19 outbreaks are described by the controlled-SIR model to an remarkable degree of accuracy. For the analysis presented in Fig. 1b,c we divided, as described in the Methods section, the official case counts by the nominal population size of the respective region or country. Seven-day centered averages are performed in addition. The country- and region-specific XI representations are then fitted by Eq. (3). The fact that the outbreaks are well described by the model, independently of the size of the country, region or city, evidences the applicability of the controlled-SIR model.

It has been widely discussed that official case counts are affected by a range of factors, which include the availability of testing facilities and the difficulty to estimate the relative fraction of unreported cases^38,39^. For example, as of mid-March 2020, the degree of testing for COVID-19, as measured by the proportion of the entire population, varied by a factor of 20 between the United States (340 tests per million) and South Korea (6100 tests per million)^40^. The true incidence might be, according to some estimates^41^ higher by up-to a factor of ten than the numbers reported in the official statistics as positive.

Case counts enter the XI representation in both the $x-$ and $y-$ axis. Scaling both *I* and *X* with a constant factor allows therefore to compensate for the undercounting problem. At the same time the control strength $\alpha _X$ needs to be rescaled, a procedure implicitly implemented for the fits shown in Fig. 1b,c. The XI framework is in this sense robust. Renormalization becomes however invalid if the undercounting of infection cases changes abruptly at a certain point during the epidemics, f.i. as a result of substantially increased testing. We will come back to this point further below. A fundamental change in the strategy followed by the government, e.g. from laissez faire to restrictive, would lead likewise to a change in $\alpha _X$, which is not captured in the current framework.

In the analysis presented in Fig. 1 daily case counts were taken as proxies for the number (relative fraction), of infected individuals $I=I(t)$. This assumption holds only up to a rescaling factor, which implies that the $g_0$ extracted for a given country or region is not the native, but an effective reproduction factor. To see this consider, e.g., the initial slope, $I\sim X(g_0-1)/g_0$, as given by Eq. (15). Rescaling daily case counts in order to obtain estimates for the number of infected individuals changes the slope and hence $g_0$. Given that the appropriate rescaling of daily case counts can only be estimated, and that we are interested here in a simple but accurate effective modeling of COVID-19 outbreaks, and not in the extraction of the native reproduction factor, we did not pursue this route.

In Table 1 we present for a number of countries and regions the obtained effective growth factors $g_0$ and the corresponding doubling times $\tau _2$, where $\tau _2=\log (2)/\log (g_0)$ defines the number of time units $\tau$ needed to double case numbers. As expected, according to the description above, one finds that the values of $g_0$ are substantially lower than the consensus estimates 2-3 for the native reproduction number^42–46^. The observed doubling times $\tau _2$ are however retained when adapting the effective time scale $\tau$ accordingly.

For a robustness check we evaluated the parameters of the controlled-SIR model assuming that only a fraction *f* of the nominal population of the country or region in question could be potentially infected, possibly due to the presence of social or geographical barriers to the disease spreading. Only marginal differences were found for $f=1/3$. The data presented in Table 1 suggest most countries followed in the first wave of the COVID-19 pandemic strict containment policies, as measured in terms of the CEI index. This insight is of particular relevance for the discussion of the costs incurring for the various containment strategies presented further below.

### Data collapse for COVID-19

Given that the XI representation is determined solely by two quantities, $X_{\mathrm{peak}}$ and $I_{\mathrm{peak}}$, universal data collapse can be attained by plotting field data normalized with regard to the respective peak values, viz by plotting $I/I_{\mathrm{peak}}$ as a function of $X/X_{\mathrm{peak}}$. It is remarkable, to which degree the country- and region specific official case counts coincide in relative units, see Fig. 1c. It implies that the controlled-SIR model constitutes a faithful phase-space representation of epidemic spreading subject to socio-political containment efforts.

**Table 1:** COVID-19 containment efficiency index.

Location		$g_0$	$\tau _2$	CEI
Italy	ITA	1.17	4.4	0.991
Iceland	ISL	1.19	4.0	0.983
Bergamo	ITA	1.20	3.8	0.972
Roma	ITA	1.20	3.8	0.998
Germany	DEU	1.21	3.6	0.995
United States	USA	1.22	3.5	0.994
Spain	ESP	1.23	3.3	0.990
Luxembourg	LUX	1.28	2.8	0.988
Austria	AUT	1.30	2.6	0.997
Israel	ISR	1.30	2.6	0.997
Australia	AUS	1.32	2.5	0.999
South Korea	KOR	1.46	1.8	1.000

For selected countries/ regions, key COVID-19 parameters, as extracted from the respective official case counts. Given is the dimensionless reproduction factor $g_0$, the doubling time $\tau _2=\log (2)/\log (g_0)$, in units of $\tau$, and the containment efficiency index $\text{ CEI }=\alpha _X/(g_0+\alpha _X)$. Note that $g_0$ is not the native, but an effective reproduction factor.

### Asymmetry of up/down time scales

For the controlled SIR model an explicit analytic expression for the $X-I$ phase space representation can be derived, as given by Eq. (3), but not for the complete timeline *X*(*t*) and *I*(*t*). Exploiting the fact that case counts are generally small with respect to the population for real-world epidemic outbreaks, the universal relation

6 $$\begin{aligned} \frac{\text{ time } \text{ down } \text{ from } \text{ the } \text{ peak }}{\text{ time } \text{ up } \text{ to } \text{ the } \text{ peak }} = 2g_0-1 \end{aligned}$$

between the time the outbreak needs to retreat from the peak, and to reach it in first place, can however be found, as shown in the Methods section. Interestingly, the ratio of down-/ and up-times is independent of the control strength $\alpha _X$ (if and only if $X\ll 1$), which suggests that Eq. (6) is valid for epidemic outbreaks in general. For COVID-19, typical values of the effective $g_0$ are of the order of 1.2-1.3, as listed in Table 1, which implies that outbreaks take of the order of 40-60% longer to retreat than to ramp up.

### Containment efficiency index

The control strength $\alpha _X$ enters the reproduction factor as $\alpha _X X$, see Eq. (2). Data collapse suggest that regional and country-wise data is comparable on a relative basis. From $\alpha _X X=(\alpha _X X_{\mathrm{peak}})(X/X_{\mathrm{peak}})$ it follows that $\alpha _X X_{\mathrm{peak}}=\alpha _X(g_0-1)/(g_0+\alpha _X)$ is a quantity that measures the combined efficiency of socio-political efforts to contain an outbreak. Dividing by $g_0-1$ results in a normalized index, the ‘Containment Efficiency Index’ (CEI):

7 $$\begin{aligned} \text{ CEI } = \frac{\alpha _X X_{\mathrm{peak}}}{g_0-1} = \frac{\alpha _X}{g_0+\alpha _X}\,, \end{aligned}$$

with $\text{ CEI }\in [0,1]$. The index is unbiased, being based solely on case count statistics, and not on additional socio-political quantifiers. Our estimates are given in Table 1. The values for the evaluated regions/ countries are consistently high, close to unity, the upper bound, indicating that the near-to-total lockdown policies implemented by most countries have been effective in containing the spread of COVID-19. A somewhat reduced CEI value is found for the particularly strongly affected Italian region of Bergamo. For South Korea the CEI is so high that its deviation from unity cannot be measured with confidence.

**Figure 3:**
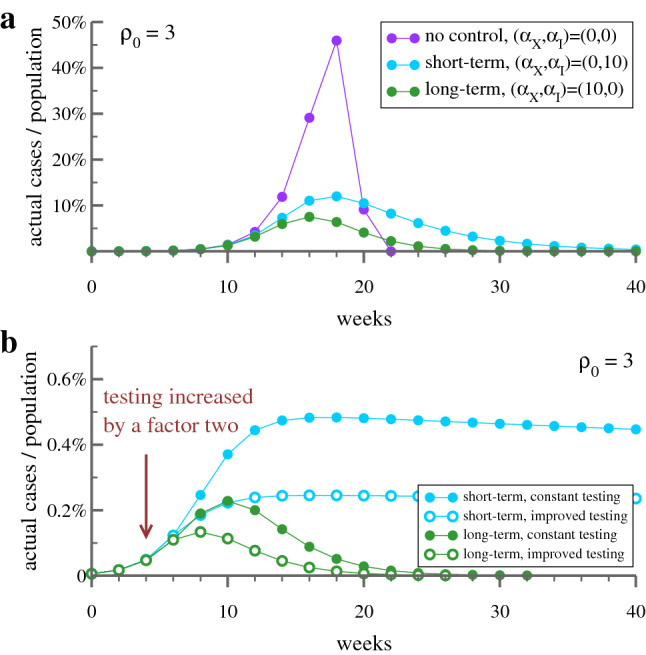
Control of epidemic peak. (**a**) Shown is the timeline of actual infected cases during an epidemic outbreak with an intrinsic reproduction factor of $\rho _0=3.0$ defined in the discrete model, which is close to COVID-19 estimates^47^. The simulation is obtained by iterating Eq. (9), with one iteration corresponding to two weeks, taken as the average duration of the illness. Short-term control, when responding to the actual number of cases, see Eq. (8), is able to reduce the peak strain on the hospital system, but only by prolonging substantially the overall duration. Long-term control, which takes the entire history of the outbreak into account, is able to reduce both the peak and the duration of the epidemic. (**b**) Increasing testing by a factor two (arrow), reduces the undercounting factor which increases, in turn, the effective response strength for both, the peak number of actual cases and the duration of the outbreak. Here $(\alpha _X,\alpha _I)=(400,0)\,/\,(0,400)$ has been used respectively for long- / short-term control.

### Long-term versus short-term control

So far, in Eq. (2) it was assumed that society and policy makers react to the total case count of infected *X*. This reaction pattern, which one may denote as ‘long-term control’, describes field data well. It is nevertheless of interest to examine an alternative, short-term control:

8 $$\begin{aligned} g = \left\{ \begin{array}{lcl} g_0/(1+\alpha _I I) &{}&{} \text{(short-term) }\\ g_0/(1+\alpha _X X) &{}&{} \text{(long-term) }\\ \end{array}\right. \end{aligned}$$

For short-term control the relevant yardstick is given by the actual case number of infected *I*. In reality, people will react to officially reported case counts, which are affected by the undercounting problem. For the terms $\alpha _I I$ and $\alpha _X X$ in Eq. (8) this corresponds to a renormalization of reaction parameters $\alpha _I$ and $\alpha _X$.

**Figure 4:**
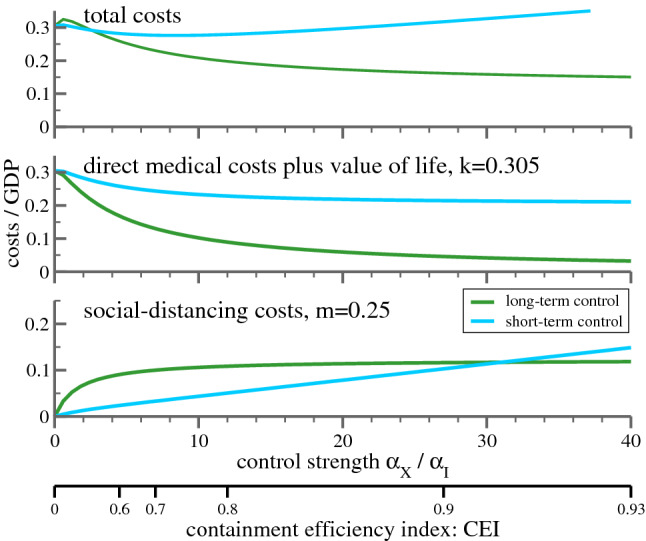
Cost of epidemic control strategies including value of life. Shown are the costs in terms of GDP$_{\mathrm{p.c.}}$, for long-term and short-term control, as defined by Eq. (8), both as a function of $\alpha _X$ and the CEI values (7), as indicated by the additional axis at the bottom. Given are the costs incurring from social distancing, Eq. (10) with $m=0.25$ (lower panel), the pure medical costs with value of life costs (middle panel), and the sum of social and medical costs (upper panel). It is assumed that the containment policy switches from mass control to individual tracking when the fraction of actual cases $I_t$ drops below a threshold of $I_{\mathrm{min}}=10^{-5}$. The starting $I_0=2\cdot 10^{-5}$.

Both control types, short- and long-term, can be employed either for the continuous-time SIR model, Eq. (1), or for the discrete-time variant,

9 $$\begin{aligned} I_{t+1} = \rho _t I_t (1-X_t), \quad \quad X_t = \sum _{k=0}^{\infty } I_{t-k}\,, \end{aligned}$$

The time-dependent reproduction factor has been denoted here as $\rho _t$, in order to make clear that discrete times are used. Short- and long-term control is then equivalent to $\rho _t=\rho _0/(1+\alpha _I I)$ and $\rho _t=\rho _0/(1+\alpha _X X)$. One time step corresponds for the discrete-time SIR model to the mean infectious period.

The simulations of Eq. (9) presented in Fig. 3 illustrate the capability of short-term and long-term reaction policies to contain an epidemic. While both strategies are able to lower the peak of the outbreak with respect to the uncontrolled ($\alpha _X=\alpha _I=0$) case, the disease will become close to endemic when the reaction is based on the actual number of cases, $I_t$, and not on the overall history of the outbreak.

Also included in the lower panel of Fig. 3 is a protocol simulating an increase of testing by a factor of two. Here $(\alpha _X,\alpha _I)=(400,0)$ and $(\alpha _X,\alpha _I)=(0,400)$ have been used as the starting reaction strengths, respectively for long- and short-term control, which are increased by a factor of two when testing reduces the undercounting ratio by one half. One observes that long-term control is robust, in the sense that increased testing contributes proportionally to the containment of the outbreak. Strategies reacting to daily case number are in contrast likely to produce an endemic state.

The framework developed here, Eqs. (1) and (2), describes mass control strategies, which are necessary when overly large case numbers do not allow to track individual infections. The framework is not applicable once infection rates are reduced to controllable levels by social distancing measures. The horizontal ’tail’ evident in the data from South Korea in Fig. 1b can be taken as evidence of such a shift from long-term mass control to the tracking of individual cases.

### Costs of controlling the COVID-19 pandemic

As shown above, the controlled-SIR model allows for a faithful modeling of the entire course of an isolated outbreak. We apply it now to investigate how distinct policies and societal reaction patterns, as embedded in the parameter $\alpha _X$, influence the overall costs of the epidemic. This is an inter-temporal approach since the cost of restrictions today to public life (lockdowns, closure of schools, etc.) must be set against future gains in terms of lower infections (less intensive hospital care, fewer deaths). Four elements dominate the cost structure: (i) The working time lost due to an infection, (ii) the direct medical costs of infections, (iii) the value of life costs, and (iv) the cost related to ‘social distancing’. The first three are medical or health-related. All costs can be scaled in terms of GDP per capita (GDP$_{\mathrm{p.c.}}$). This makes our analysis applicable not only to the US, but to most countries with similar GDP$_{\mathrm{p.c.}}$, e.g. most OECD countries.

### Overall cost estimates

The cost estimates, which are given in detail in the Supplementary Information, can be performed disregarding discounting. With market interest rates close to zero and the comparatively short time period over which the epidemic plays out, a social discount rate between 3% and 5% would make little difference over the course of one year^48^.

Total health costs $C^{\mathrm{medical}}$ incurring over the duration of the epidemic are proportional to the overall fraction $X_{\mathrm{tot}}=X_{t\rightarrow \infty }$ of infected, with a factor of proportionality *k*. We hence have $C^{\mathrm{medical}} =kX_{tot}$. We estimate $k\approx 0.305$ in terms of GDP$_{\mathrm{p.c.}}$ when all three contributions (working-time lost, direct medical cost, value of life) are taken into account, and $k\approx 0.14$ when value of life costs are omitted.

The economic costs induced by social-distancing measures, $C^{\mathrm{social}}$, depend in a non-linear way on the evolution of new cases (short-term control) or the percentage of the population infected (long-term control). To be specific, we posit that the reduction of economic activity is percentage-wise directly proportional to the relative reduction in the reproduction factor^49^, viz to $(\rho _0-\rho _t)/\rho _0$:

10 $$\begin{aligned} C^{\mathrm{social}} = \sum _{I_{t}>I_{\mathrm{min}}} \text{ c}_t^{\mathrm{s}}, \quad \quad \text{ c}_{t}^{\mathrm{s}} = m\ \frac{\rho _0-\rho _t}{\rho _0}\,\frac{2}{52}\,, \end{aligned}$$

where 2/52 is the per year fraction of 2-week quarantine period. The epidemic is considered to be under control when the fraction of new infections $I_t$ falls below a minimal value $I_{\mathrm{min}}$. As detailed out in the Supplementary Information, a comprehensive analysis yields $m\approx 0.25$ in terms of GDP$_{\mathrm{p.c.}}$. Note that the ansatz Eq. (10) holds only when mass control is operative, viz when large case numbers do not allow the tracking of individual infections.

**Figure 5:**
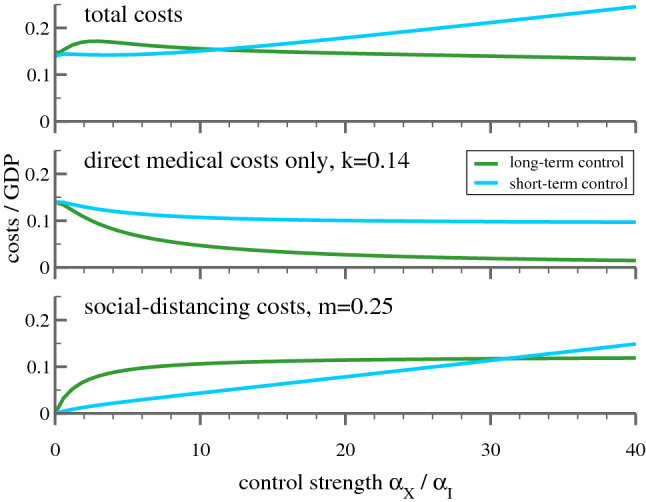
Cost of epidemic control without value of life. As in Fig. 4 (bottom panels are identical), but without the value of life costs. A long-term strategy with intermediate reaction strength is costlier than a hands-off policy.

Once *k* and *m* are known, one can compare the total costs incurring as the result of distinct policies by computing the sum of future costs for different values for $\alpha _X$ in Eq. (2). This is illustrated in Fig. 4 with the value of life costs included ($k=0.305$), and in Fig. 5, without value of life costs ($k=0.14$). Given are the total cumulative costs for the two strategies considered, long-term and short-term control, both as a function of the respective implementation strength, as expressed by the value of $\alpha _X$ and $\alpha _I$.

The middle panel of Fig. 4 shows that a society focused on short-term successes will incur substantially higher medical costs, because restrictions are relaxed soon after the peak. By contrast, if policy (and individual behavior) is influenced by the total number of all cases experienced so far, restrictions will not be relaxed prematurely and the medical costs will be lower for all values of $\alpha _X$. The bottom panel shows the social distancing costs as a fraction of GDP$_{\mathrm{p.c.}}$, which represent a more complicated trade-off between the severity of the restrictions and the time they need to be maintained. If neither policy, nor individuals react to the spread of the disease ($\alpha _X=0$) the epidemic will take its course and costs are solely medical. This changes as soon as society reacts, i.e. as $\alpha _X$ increases. Social distancing costs increase initially (i.e. for small values of $\alpha _X$), somewhat stronger for the long-term than for the short-term reaction framework. The situation reverses for higher values of $\alpha _X$ and $\alpha _I$ with $\alpha _X, \alpha _I \approx 30$ being the turning point. From there on, the distancing cost from a long-term based reaction falls below that of the short-term strategy. The sum of the two costs is shown in the uppermost panel. For large values of $\alpha _X$, $\alpha _I$ short-term policies result in systematically higher costs.

Supplementary Figure 1 of the Supplementary Information shows that short-term control cannot explain observed COVID-19 outbreaks per se. Our estimates for the incurring costs suggest that economic cost considerations may have caused countries to follow predominantly long-term control strategies during the first wave of the COVID-19 outbreak.

## Discussion

**Figure 6:**
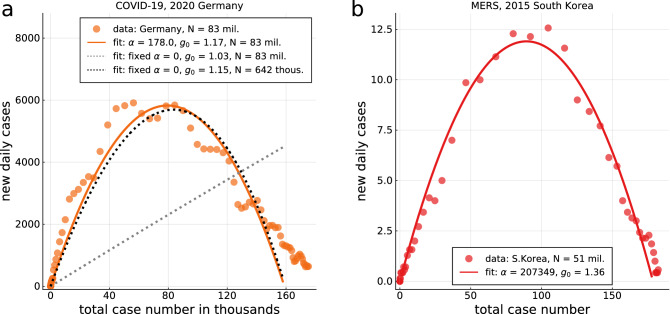
Case count modelling. (**a**), Modeling case counts as uncontrolled outbreaks. Case counts, here for Germany (seven-day centered averages, dots), can be modeled using either the full XI representation (full line), as given by Eq. (3), or with the standard uncontrolled SIR model ($\alpha _X=0$, dashed lines). Using the nominal population size for Germany, 83 Million, leads to an utterly unrealistic $\alpha _X=0$ curve (dashed, grey). The best $\alpha _X=0$ fit is obtained when a fictitious population size of 478 Thousand is assumed (dashed, black). An epidemic abates on its own only when the population size is of the order of the total case count divided by $X_{\mathrm{tot}}$. (**b**) XI representation of the 2015 MERS outbreak in South Korea, covering a total of 186 cases. A $n=7$ centered average has been used, in view of the small case numbers.

The total costs of competing containment strategies can be estimated if the feedback of socio-political measures can be modeled. For this one needs two ingredients: (i) a validated epidemiological model and (ii) a link between the impact of containment efforts, in terms of model parameters, to their economic costs. Regarding the first aspect, we studied the controlled-SIR model and showed that COVID-19 outbreaks follow in many cases the phase-space trajectory, the XI representation, predicted by the analytic solution. The same holds for the 2015 MERS outbreak in South Korea, as shown in Fig. 6b. We extracted for a number of countries and regions estimates for the intrinsic doubling times and found that they are not correlated to the severity of the outbreak. Regarding the second aspect, we proposed that the economic costs of social distancing are proportional to the achieved reduction in the infection rate^49^. Equation (10) establishes the required link between epidemiology, political actions and economic consequences. Health-related costs, which are related to official case counts, are in contrast comparatively easier to estimate. We have not considered formally the optimal control problem, which would consist of minimizing the sum of total costs if the control strength could be chosen freely for every period. Instead, we have been interested here in comparing distinct containment strategies under which society and governments react in a predictable pattern to the spread of the disease.

A non-trivial outcome of our study is that strong suppression strategies lead to lower total costs than taking no action, when containment efforts are not relaxed with falling infection rates. A short-term control approach of softening containment with falling numbers of new cases is likely to lead to a prolonged endemic period. With regard to the ‘exit strategy’ discussion, these findings imply that social distancing provisions need to be replaced by measures with comparative containment power. A prime candidate is in this regard to ramp up testing capabilities to historically unprecedented levels, several orders of magnitude above pre-Corona levels. The epidemic can be contained when most new cases are tracked, as implicitly expressed by the factor $\alpha _X$. This strategy can be implemented once infection rates are reduced to controllable levels by social distancing measures. Containment would benefit if the social or physical separation of the ‘endangered’ part of the population from the ‘not endangered’ would be organized in addition on a country-wide level, as suggested by community-epidemiology. With this set of actions the vaccine-free period can be bridged.

As a last note, there is a sometimes voiced misconception regarding the meaning of the herd immunity point, which occurs for an infection factor of three when 66% of the population is infected. Beyond the herd immunity point, the infected-case counts remain elevated for a considerable time. The outbreak stops completely only once 94% of the population has been infected, as illustrated in Fig. 1a.

## Methods

### Validation of the model from COVID-19 data

In Fig. 2 we show how the model given in Eq.  (2) is validated by COVID-19 data. Fig. 2a displays the collected data of infected population during the first wave of the COVID-19 pandemic in a range of representative countries and regions. Plotted is the time-dependent reproduction factor R$_t$ as a function of the relative cumulative case count $X / X_{\mathrm{peak}}$. We followed standard procedures^37^ and defined $R_t$ as the fraction of newly infected individuals at time *t* with respect to the infected individuals at time $t-4$ days, $R_t = \overline{I}_t / \overline{I}_{t-4}$, where seven-day centered moving averages $\overline{I}_t = \sum _{s=t - 3}^{t+3} I_s$ are considered. Also shown is a fit to the data using the functional form predicted by our model, Eq. (2). The quantitative comparison between field data and modeling validates the controlled-SIR model. For a set of representative countries and regions it is shown in Fig. 2b that there is a direct correlation between the measured reproduction factor $R_t$ and the effective reproduction factor $g_t$, as defined by Eq. (2).

### Data collection and handling

Data has been accessed as of May 18 (2020) via the public COVID-19 Github repository of the Johns Hopkins Center of Systems Science and Engineering^50^. Preprocessing was kept minimal, comprising only a basic smoothing with sliding averages. If not stated otherwise, a seven day centered average (three days before/after, plus current day) has been used. Robustness checks with one, three and five day sliding averages were performed, as shown in Fig. 1d. Fractional case counts are obtained by dividing the raw number by the respective population size. For the case of South Korea, the XI-analysis was performed using the initial outbreak, up to March 10 (2020). China has been ommitted in view of the change in case count methodolgy mid February 2020.

The variable *I* represents in the SIR model the fraction of the population that is infectious, which for this model coincides with the infected population. For the COVID-19 data, we used instead an XI-representation for which the number of new daily cases is plotted against the total case count. This procedure is admissible as long as the relative duration of the infectious period does not change.

### Fitting procedure

We compared the theoretical result for the controlled SIR model, $I(X)\equiv I^{\mathrm{(theory)}}(X)$, see Eq. (3), to the reported data $I_t^{\mathrm{(data)}}$, where *t* runs over all days. The field data $X_t^{\mathrm{(data)}}$ for the total case number is crowded at low levels of *X* and *I* in the XI representation. A fitting procedure that takes the range $X\in [0,X_{\mathrm{tot}}]$ uniformly into account is attained when minimizing the weighted loss function

11 $$\begin{aligned} U = \sum _t u_t\left( I_t^{\mathrm{(data)}}-I^{\mathrm{(theory)}}(X_t^{\mathrm{(data)}})\right) ^2\,. \end{aligned}$$

For the weight we used $u_t=X_t^{\mathrm{(data)}}-X_{t-1}^{\mathrm{(data)}}= I_t^{\mathrm{(data)}}$, which satisfies the sum-rule $\sum _t u_t=X_{\mathrm{tot}}$, where $X_{\mathrm{tot}}$ is the total (maximal) case count. With Eq. (11) it becomes irrelevant where the timeline of field data is truncated, both at the start or at the end. Adding a large number of null measurements after the epidemic stopped would not alter the result. Numerically the minimum of *U* as a function of $g_0$ and $\alpha _X$ is evaluated.

### Modeling field data as uncontrolled outbreaks

It is of interest to examine to which degree official case statistics could be modeled using an uncontrolled model, $\alpha _X=0$. For this purpose it is necessary to assume that the epidemics stops on its own, which implies that one needs to normalize the official case counts not with respect to the actual population, but with respect to a fictitious population size *N*. In this view the outbreak starts and ends in a socially or geographically restricted community. The results obtained when optimizing *N* are included in Fig. 6a. At first sight, the $\alpha _X=0$ curve tracks the field data. Note however the very small effective population sizes, which are found to be 478000 for the case of Germany. Alternatively one may adjust $g_0$ by hand during the course of an epidemic, as it is often done when modeling field data.

### Analytic solution of the controlled-SIR model

Starting with the expression for the long-term control, Eq.  (2), one can integrate the controlled-SIR model Eq. (1) to obtain a functional relation between *S* and *I*. Integrating $\dot{I}/\dot{S}$, viz

$$\begin{aligned} dI = -dS +\frac{1}{g(S)S}\,dS = -dS + \frac{1}{g_0}\,\frac{1+\alpha _X(1-S)}{S}\,dS\,, \end{aligned}$$

yields

12 $$\begin{aligned} I = -\left( \frac{\alpha _X}{g_0}+1\right) S+ \frac{1+\alpha _X}{g_0}\,\log (S) +c\,, \end{aligned}$$

where the integration constant *c* is given by the condition $I(S\!=\!1)=0$. Substituting $S=1-X$ one obtains consequently the XI-representation Eq. (3). The analogous result for $\alpha _X=0$ has been derived earlier^51^. The number of actual cases, *I*, vanishes both when $X=0$, the starting point of the outbreak, and when the epidemic stops. The overall number of cases, $X_{\mathrm{tot}}$, is obtained consequently by the non-trivial root $X_{\mathrm{tot}}$ of Eq. (3), as illustrated in Fig. 1a. As a side remark, we mention that the XI representation allows us to reduce Eq. (1) to

13 $$\begin{aligned} \tau \dot{S}= -\frac{gS}{g_0}\Big [ (\alpha _X+g_0)(1-S)+(1+\alpha _X)\log (S) \Big ]\,, \end{aligned}$$

which is one dimensional. Integrating Eq. (13) with $g=g(S)$ yields $S=S(t)$, from which *I*(*t*) follows via $\tau \dot{I} = \big (gS -1\big )I$ and *R*(*t*) from the normalization condition $S+I+R=1$.

### Large control limit of the XI representation

Expanding Eq. (3) in *X*, which becomes small when $\alpha _X\gg 1$, one obtains

14 $$\begin{aligned} I=\frac{1+\alpha _X}{2g_0}X\left[ 2\,\frac{g_0-1}{1+\alpha _X}-X\right] + O(X^3)\,, \end{aligned}$$

which makes clear that the phase-space trajectory becomes an inverted parabola when infection fractions are small. As a consequence one finds

15 $$\begin{aligned} I \approx \frac{g_0-1}{g_0}\,X + O(X^2)\,, \end{aligned}$$

which shows that the slope $dI/dX=(g_0-1)/g_0$ at $X\rightarrow 0$ is independent of $\alpha _X$ and of the normalization procedure used for *I* and *X*. The first result was to be expected, as $\alpha _X$ incorporates the reaction to the outbreak, which implies that $\alpha _X$ contributes only to higher order. The dimensionless natural growth factor $g_0$ is hence uniquely determined, modulo the noise inherent in field data, by measuring the slope of the daily case numbers with respect to the cumulative case count.

From Eq. (14) one obtains

16 $$\begin{aligned} X_{\mathrm{tot}}\big |_{\alpha _X\gg 1} \approx 2\,\frac{g_0-1}{\alpha _X}\, \end{aligned}$$

for the total number of infected $X_{\mathrm{tot}}$ in the large-control limit. In analogy one finds

17 $$\begin{aligned} I_{\mathrm{peak}}\big |_{\alpha _X\gg 1}\approx \frac{(g_0-1)^2}{g_0\alpha _X}, \quad \quad X_{\mathrm{tot}} \approx \frac{2g_0}{g_0-1}\,I_{\mathrm{peak}} \end{aligned}$$

from Eq. (3), and in comparison with Eq. (16).

### Time scale asymmetry

From the one-dimensional representation (13) of the controlled SIR model one can estimates two characteristic time scales. For this purpose one considers an initial relative infection status $f_X X_{\mathrm{tot}}$, with $f_X>0$ and $f_X\ll 1$.

- *Run-up* $T_{\mathrm{up}}$, defined as the time needed to reach the peak when starting from $X_{\mathrm{start}}=f_X X_{\mathrm{tot}}$.
- *Run-down* $T_{\mathrm{down}}$, defined as the time needed to reach $X_{\mathrm{end}}=(1-f_X) X_{\mathrm{tot}}$, down from the peak.

In general one needs to integrate Eq. (13) numerically. Given that real-world fractional case counts *X* are small, $X< X_{\mathrm{tot}}\ll 1$, one can simplify (13), as for (14), obtaining

18 $$\begin{aligned} t-t_0 = \frac{\tau }{g_0-1}\log \left( \frac{X}{(X_{\mathrm{tot}}-X)^{2g_0-1}}\right) \,. \end{aligned}$$

It follows directly that $T_{\mathrm{down}}/T_{\mathrm{up}}=2g_0-1$, as stated in Eq. (6). For a pathogen to spread its dimensional growth factor $g_0$ needs to be larger than unity, compare Table 1. Going down takes hence substantially longer than ramping up.

## Supplementary Information

Supplementary information.

## Data Availability

The COVID-19 data examined is publicly accessible via the COVID-19 Github repository of the Johns Hopkins Center of Systems Science and Engineering https://github.com/CSSEGISandData/COVID-19. Data for the 2015 MERS outbreak in South Korea is publicly available from the archive of the World Health organization (WHO), https://www.who.int/csr/disease/coronavirus_infections/archive-cases/en/.
