# Supplementary material for: Containment efficiency and control strategies for the corona pandemic costs
Source: Sci Rep. 2021 Mar 25;11:6848. doi: 10.1038/s41598-021-86072-x (PMC7994626; doi:10.1038/s41598-021-86072-x)
Supplement: Supplementary file 1 — Supplementary information. [file 41598_2021_86072_MOESM1_ESM.pdf]

SUPPLEMENTARY INFORMATION
Containment efficiency and control strategies for the Corona pandemic costs

Claudius Gros,[1,*] Roser Valenti,[1] Lukas Schneider,[1] Kilian Valenti,[2] and Daniel Gros[3,4]

[1] *Institute of Theoretical Physics, Goethe University, 60438 Frankfurt a.M., Germany*
[2] *Vivantes Klinikum Spandau, 13585 Berlin, Germany*
[3] *Department of Economics, University of California, Berkeley, USA*
[4] *CEPS (Centre for European Policy Studies), 1000 Brussels, Belgium*

(Dated: January 4, 2021)

Societal reaction to the spread of the disease

The two strategies investigated here, short-term and long-term, correspond to reaction patterns that are observed for the COVID-19 outbreak[1,2]. In Supplementary Figure 1 we present some examples. For both cases, the societal reaction described by the parameter α_X and α_I can be thought as a sum of two contributions

$$\alpha_X = \alpha_s + \alpha_g, \qquad (1)$$

where α_s quantifies the spontaneous reaction by the population and α_g encodes government interventions. Analogously, such a sum of two contributions can be made for α_I.

The first contribution, α_s, takes into account societal behavioral changes happening when a substantial fraction of the population spontaneously adopts social distancing (avoiding hand-shakes, restaurants, cinemas, etc.), f.i. in response to media reports about the severity of the outbreak. Voluntary social distancing can lead to substantially reduced restaurants and cinemas attendances even before governments impose mandatory school closures, curfews and other drastic measures.[3] An important aspect of the spread of COVID-19 is the distinct reactions of societies in different countries. In Asia the wearing of masks becomes a convention, which is likely to correspond to a higher α_s, while such a measure tends to be resisted by a majority of European populations.[4]

The second contribution to the control factor, α_g, captures the role of government interventions. Measures ranging from forbidding large events, to school closures and, finally, to lockdowns, become politically possible when the number of individuals infected increases and surpasses critical levels.

The aim of our investigation is not to evaluate the effectiveness of specific measures, which has been done elsewhere[5,6], but to assess the dynamical effect of the societal reaction encoded in the feedback parameter α_X, on the overall evolution of the epidemic. As most social distancing measures are costly, both for the economy and overall well-being[7], it is reasonable to assume that their strictness is increased only when necessary, viz in relation to the severity of the outbreak. The latter can be measured either by the number of current cases, I_t, or by the cumulative case count X_t.

The inverted U shape of the total cost of the virus as a function of α_X has one important corollary: the 'laissez faire' equilibrium is not the optimum for society. If the government abstains from action leaving it to societal reaction to dampen the peak, the spread of the disease would be limited only by α_s, which might bring society close to the hump of the total cost curve. Strong government action, i.e. a high value of α_g could then push the path to the other side of the hump resulting in lower costs. In other words, relying only on individual reaction which aims at lowering the risk to oneself, would be sub-optimal. This is of course a general result for all contagious diseases[8,9] but we confirm it accounting explicitly for the cost of the measures needed to protect public health.

Detailed costs of controlling the COVID-19 pandemic

In what follows we present a detailed estimation of the costs of controlling the COVID-19 pandemic given in GDP per capita ($\mathrm{GDP}_{\mathrm{p.c.}}$) to ensure comparability across countries.

Four elements dominate the cost structure: (i) The working time lost due to an infection, (ii) the direct medical costs of infections, (iii) the value of life costs, and (iv) the cost related to 'social distancing'. The first three are medical or health-related.

Health costs, loss of working time

A first direct impact of a wave of infections is that a fraction of the population cannot work. Based on the Diamond Princess data[10], where the entire population was tested, we estimate that only half of the infected develop symptoms that require them to stay home for a one- to two-week period and an additional two-week period until they are no longer contagious. About 20% of the infected (or 40% of those with symptoms) develop stronger symptoms requiring one additional period of absence from work[11]. To be conservative, we assume that there are no severe cases or deaths among the working age. This results in a reduction in the work force per year (52 weeks) of around $(0.3 \times 2 + 0.2 \times 3) \times (2/52) = 2.4/52 = 0.05$, for every 1% of the population infected.

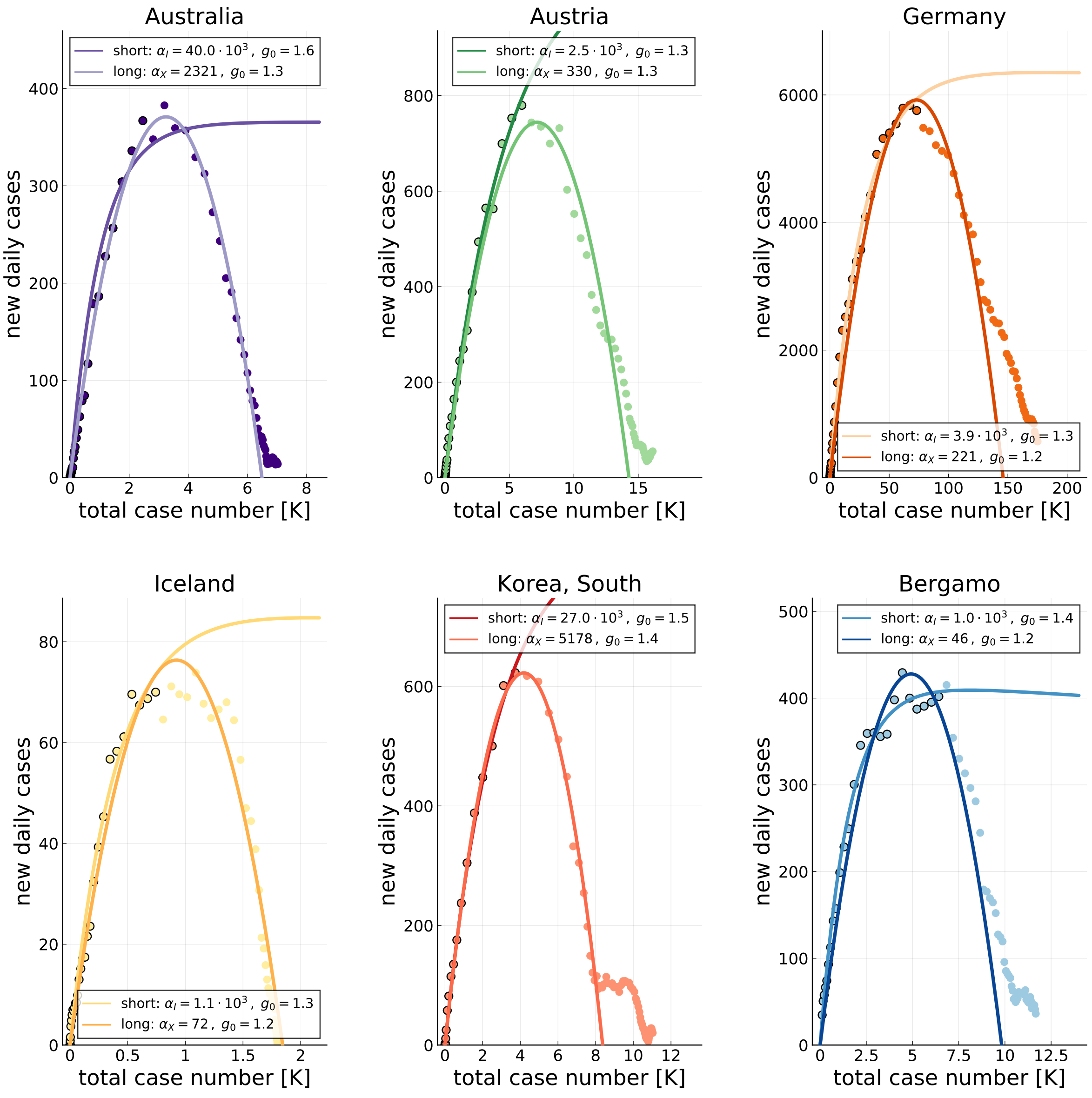

Supplementary Figure 1. | **Short- vs.- long-term containment.** Fits to COVID-19 data for short-term control, $(\alpha_I, \alpha_X) = (\alpha_I, 0)$ and, for comparison, for long-term control, $(\alpha_I, \alpha_X) = (0, \alpha_X)$. The parameter value are given in the respective legends. The intial slope, which reflect the initial phase of exponential growth, has been taken as a reference.

Medical costs, treatment, hospitalization

Hospitalization rates and costs of hospital treatment for COVID-19 vary enormously across across countries. But it is estimated that about 20%[12] of the infected individuals require some sort of hospitalization. A recent large scale survey of the literature[13] shows that about one fourth of them (around 5% of the infected) need intensive care and roughly on sixth dying. We adopt a more conservative fatality rate among the hospitalized of 1%[10].

As a comparison, we note that an average influenza season leads to a hospitalization of about 0.12% of the US population[14]; and one fourth of them require intensive care, with one twentieth (0.13% of all infected) dying[10]. Averaged over the 2010-17, of the order of 35 thousand influenza-related deaths per year have been registered in the US, one tenth of the over 300 thousand COVID-19 related fatalities registered in 2020.

Costs: Intensive care with ventilation is the most costly form of life saving in hospital care[13]. In the US, the cost of two weeks of an intensive care unit is equivalent to about 1 year (100%) of $\mathrm{GDP}_{\mathrm{p.c.}}$[15]. For Covid-19 patients in the US, it has been reported that average hospitalisa-

tion costs per case amount to over 70 thousand USD, or 115% of (annual) GDP per capita[16].

In Germany, which might be typical of the rest of Europe, the cost of two weeks of intensive care appears to be lower, around 20,000 Euro, or roughly 60% of $\mathrm{GDP}_{\mathrm{p.c.}}$[17]. We use the German parameters for a conservative estimate of medical costs. The cost of general hospitalization for two weeks is assumed to be 12,000 Euro, and equivalent to about 30% of $\mathrm{GDP}_{\mathrm{p.c.}}$. With two weeks of general hospitalization and two weeks of intensive care for severe cases, this results in a medical cost of $(0.05 \times 0.6 + 0.05 \times 0.3 + 0.15 \times 0.3 = 0.09)$, that is of 9% $\mathrm{GDP}_{\mathrm{p.c.}}$.

Value of lives lost

Third, the cost of premature death through the disease represents the most difficult element to evaluate in financial terms. We will show below that our central results remain valid even without assigning a monetary value to lives lost, but since major contributions[18] are based on an evaluation of the economic value of lives lost, we show how this point can be incorporated into our framework. There are two ways to attribute a monetary value on a life saved or lost. The first, mentioned in the main text, is based on the concept of a Value of Statistical Life (VSL), which is commonly used in the impact assessment of public policy that aims at lowering the probability of a premature death[19]. A typical application scenario for VSL is the case when the probability of death is very low (e.g. car accidents), but could be lowered even more (seat belts). For COVID-19, a high-death epidemic, we prefer a medical-based approach, which allows us to produce conservative estimates. VSL arrives in contrast often at much higher values, up to millions of Euro or Dollars[20]. Putting a monetary value on lives saved is unavoidable in medical practice that is confronted with the problem of selecting the procedures to be used to prolong life - a situation that arises for many patients infected by the Coronavirus under intensive care.

The literature dealing with the cost of medical procedures finds a central range of between 100,000 and 300,000 Dollars per year of life lost[21,22]. Given the current US $\mathrm{GDP}_{\mathrm{p.c.}}$, these values translate into a range of 1.5 to 4 years of $\mathrm{GDP}_{\mathrm{p.c.}}$. Cutler and Richardson[23] argue for a value equivalent to three times $\mathrm{GDP}_{\mathrm{p.c.}}$. We use the lower bound of this range (i.e. 1.5 times $\mathrm{GDP}_{\mathrm{p.c.}}$) for most of our simulations, which might thus underestimated the value of lives saved through social distancing restrictions.

What remains to be determined is the number of years lost when a Corona patient dies.

On the cruise ship Diamond Princess[10,24] which served almost as a laboratory, the average age at death was 76 years. Cruise passengers tend to have fewer acute health conditions than the general population, thus rendering the co-morbidity argument less prominent. The remaining life expectancy (weighted by the difference incidence by sex) would thus be 11 years. This implies that the economic value of the premature deaths should be equal to about 11 times the loss for one year of life saved (potentially higher for most European countries which tend to have a higher life expectancy).

For each 1% of the population the value of lives lost would thus be equal to $0.01 \times 11 \times$ the nominal value of one year of life.

The value of life can be measured in terms of multiples of $\mathrm{GDP}_{\mathrm{p.c.}}$, which allows to write the sum of the three types of health or medical costs (loss of working time, hospitalization and value of lives lost) as a linear function of the percentage of the population infected:

$$c_t^{\mathrm{med}} = k I_t$$

with a proportionality factor k being equal to the sum of the three contributions. Scaling k with the $\mathrm{GDP}_{\mathrm{p.c.}}$ allows for an application and comparison across countries. Using the lower bound of the central range yields then the following calibration of the medical costs:

$$(0.05 + 0.09 + 0.01 \times 1.5 \times 11) \times \mathrm{GDP}_{\mathrm{p.c.}} = 0.305 \times \mathrm{GDP}_{\mathrm{p.c.}} \qquad (2)$$

The upper bound for the value of k would be substantially higher: $(0.05 + 0.09 + 0.01 \times 4 \times 11) \times \mathrm{GDP}_{\mathrm{p.c.}} = 0.58 \times \mathrm{GDP}_{\mathrm{p.c.}}$. For the numerical calculations we will use the conservative estimate $k = 0.305$ in terms of $\mathrm{GDP}_{\mathrm{p.c.}}$.

If we only consider the direct medical costs consisting of loss of working time and hospitalization, without including the value of lives lost, the proportionality factor in equation (2) reduces to $k = 0.14$ in terms of $\mathrm{GDP}_{\mathrm{p.c.}}$.

Medical costs over the lifetime of the epidemic

The cost estimates discussed so far, c_t^{med}, refer to the per-period cost of the currently infected. For the total cost over the entire endemic we need to calculate the discounted sum of all c_t^{med} over time. Given that a period corresponds to about two weeks, we neglect discounting, which would make little difference even if one uses a social discount rate of 5% instead of using market rates (which may be negative). The total medical costs over the course of the endemic can be written as the simple sum of the cost per unit of time:

$$C^{\mathrm{medical}} = \sum_{I_t > I_{\min}} c_t^{\mathrm{med}} = k X_{tot}\,. \qquad (3)$$

The epidemic is considered to have stopped when the fraction of new infections I_t falls below a minimal value, $I_{\min}$.

Using the conservative estimate (low value of life) $k = 0.305$ it is straightforward to evaluate the total cost of a policy of not reacting at all to the spread of the disease, which would lead in the end to $X_{\mathrm{tot}} = 0.94$. A hands-off

policy would therefore lead to medical costs of over 28% of GDP.

In absolute terms the cost of a policy of doing nothing would amount to 1000 billion Euro for a country like Germany. For the US the sum would be closer to 5 Trillion of Dollars (25% of a GDP of 20 Trillion of Dollars). As it would not be possible to ramp up hospital capacity in the short time given the rapid spread of the disease, the cost would be in reality substantially higher, together with the death toll[6,18]. We abstract from the question of medical capacity (limited number of hospital beds) because we assume that society would react anyway as the virus spreads, thus limiting the peak, and, second, we are interested in the longer term implications of different strategies and not just in their impact on the short-term peak.

We note that even concentrating only on the direct medical cost and working time lost (k=0.14) a policy of letting the epidemic run its course through the entire population would lead to losses of working time and hospital treatment of over 13% of GDP (94% of 14%). By comparison, total health expenditure in most European countries amounts in normal times to about 11% of GDP[25]. Even apart from ethical considerations, to avoid or not potentially hundreds of thousands of premature deaths, there exists thus an economic incentive to slow the spread of the COVID-19 virus.

Given the somewhat contentious nature of the value of lives lost, we present in the middle panel of Fig. 4 of the main text the medical cost estimates (as a proportion of GDP) without including the value of life costs (results with including the value of life costs are shown in the main text). As shown in the figure, increasing α_X leads to a lower medical cost because the percentage of the population infected will be lower. The difference between short-term and long-term control increases for higher values of α_X. At these α_X values the medical cost over the entire endemic would be lower because the overall fraction of infected population is lower. For a strongly reactive society and policy i.e. for $\alpha_X \gg 1$ (and the case of long-term control), an explicit solution for the total health cost is given by,

$$C^{\text{medical}} = kX_{\text{tot}}\big|_{\alpha_X \gg 1} \approx 2k\,\frac{g_0 - 1}{\alpha_X} \tag{4}$$

which implies that the total health or medical costs are inversely proportional to the strength of the policy reaction parameter. Draconian measures from the start, i.e. with α_X going towards infinity, reduce the medical costs to close to zero - irrespective of whether one adds the value of lives lost. This can be seen in Fig. 3 and Fig. 4 of the main text, where the medical cost (over the entire epidemic) starts for $\alpha_X = 0$ at values close to k because without any societal reaction 94% of the population would get infected and with increasing α_X the medical costs decline monotonously.

Social distancing costs

The economic costs of imposing social distancing on a wider population are at the core of policy discussions and drive financial markets. As mentioned above, social distancing can take many forms; ranging from abstaining from travel or restaurant meals to government interventions enforcing lockdowns, quarantine, closure of schools, etc. This cost is more difficult to estimate. However, a rough estimate is possible if one takes into account that most economic activity involves some social interactions. Limiting social interaction thus necessarily reduces economic activity. This suggests that the economic cost of the social distancing described in equation (2) of the main text should increase with the reduction in the transmission rate described by g.

Without any social distancing, $\alpha_X = 0$, the economy would not be affected by the spread of the virus. Stopping all economic social interactions would bring the economy to a halt, but the reproduction rate of the virus would also go close to zero (Eichenbaum *et al.*[26] make a similar assumption). We thus posit that the (per-time unit) social-distancing economic cost c_t^{s} is proportional to the reduction in the transmission rate. The total economic costs C^{social} can be written as the sum of c_t^{s}:

$$C^{\text{social}} = \sum_{I_t > I_{\min}} c_t^{\text{s}}, \qquad c_t^{\text{s}} = m\left[1 - \frac{\rho_t}{\rho_0}\right]\frac{2}{52} \tag{5}$$

considering here the notation of the discrete-time controlled SIR model (equation (8)). The key question is the factor of proportionality, m, which links the severity of social distancing to the reduction in economic activity. Popular attention has focused on services linked directly to social contact. There exist indeed selected sectors which will completely shut down under a lockdown. However, these sectors (tourism, non-food retail, etc.) account for a limited share of the economy (less than 10% for most countries). Expenditure for food is actually little affected since even under the most severe lockdown, grocery shopping is still allowed and families must consume more food at home as they cannot go out to restaurants.

The manufacturing sector is less affected by social distancing than the service sector because in modern factories workers are scattered over a large factory floor, making it relatively easy to maintain production while maintaining the appropriate distance between workers. Moreover, some sectors, e.g. finance, can work online with only a limited effect on productivity. The widespread impression that the entire economy stops under a lockdown is thus not correct. The drastic measures adopted in China illustrate this proposition: when all non-essential social interactions were forbidden, industrial production and retail sales fell by 'only' 20-25%[27] while the reproduction factor went from 3 to 0.3, a fall by a factor of ten. Using this experience we calibrate the parameter m at 0.25. The projections of the International Monetary

Fund, a loss of output of about 8% for severe lockdowns lasting one quarter[28],[29] confirm this order of magnitude.

A reduction in the reproduction factor ρ_t to one tenth its normal epidemiological value of ρ_0 would thus lead to a loss of GDP of 25% for the time period during which the restriction or social distancing measures are in place. This would imply that an abrupt shutdown of the economy to 25% of its capacity for 12 weeks, or 6 incubation periods would cost about $0.25\times(12/52)$, or about 6% of annual GDP. A reduction of GDP by 6% represents a recession even deeper than the one which followed the financial crisis of 2009. This is compatible with current forecasts of zero GDP growth in China in 2020 (relative to a baseline of 5-6% before the crisis). But even such a large cost in terms of output foregone would be below the medical cost arising from herd immunity[30]. Even apart from ethical considerations, it would thus appear to make sense to accept a temporary shut down of parts of the economy to avoid the huge medical costs.

A first result is thus that if one compares two extremes: letting contagion run its course (herd immunity) or draconian measures, the social costs are lower in the second case. Small changes to the key parameters, k and m, might change the exact values of the costs in terms of overall magnitude, but the ranking appears robust.

We do not consider separately the fiscal cost, i.e. the cost for the government to save millions of enterprises from bankruptcy and ensure that workers have a replacement income when they get laid off. This cost to governments is a transfer within the country from one part of society (tax payers) to those who suffer most under the economic crisis.

A key issue in the discussion on the economic cost of social distancing is the question about how long these measures need to be maintained. It is sometimes argued that the cost of a policy of social distancing would be unacceptably high because the measures could not be relaxed until the virus had been totally eradicated. However, this pessimism is not warranted by the success of a strategy of 'testing and tracing' implemented in some countries (mainly those which had experienced SARS). Such a strategy is, of course, only possible if the starting number of infections is low enough to allow for individual tracing.

We thus make the assumption that when the number of active cases falls below a certain threshold, the costly measures of general social distance containment are no longer needed and can be substituted by pro-active repeated testing coupled to quick follow-up of the remaining few cases which are quarantined and whose contacts are quickly traced. In this case the resulting economic cost is assumed to fall away. The experience of Taiwan[31], Korea[32] and Japan suggests that when the infected are less than one per 100,000, general social distancing is no longer required (assuming mass testing has been adopted in the meantime so that the infections can be accurately measured).

Parameter updating

The estimates on which our results are based will have to be updated when actualized COVID-19 data is available in the future. The WHO-China Joint Mission Report suggests a ρ_0 (g_0 in the continuous-time representation) per infected of $2-2.5$[33] (in units of the disease duration), while we use the figures from Liu *et al.*[34], who predict a reproduction factor of around three. The numbers for the forecast of health costs are derived in part from the Diamond Princess data[10], for which the population was comparatively healthy. The statistics for symptoms requiring the absence from work may therefore in reality be somewhat higher.

The hospitalization and mortality rate are estimated with a substantial uncertainty, due to the high numbers of unregistered and untested infections. Early studies based on official data from China[35,36] estimated that the number of actual infections may be between 10 to 20 times higher than the number of detected infections. However, serological test in e.g. Austria suggest only a factor of 3[37]. The continuing screening of blood samples via the US, Nationwide Commercial Laboratory Seroprevalence Survey, showed in September 2020 an average undercounting factor of 2.6, as opposed to our assumption of 2. Leaving possibly lower, but still substantial true hospitalization and mortality rates for COVID-19.

A strong age gradient has been observed for the case fatality rate of COVID-19 by age[38], which could be logistic[39], and there are large variations across countries[40]. Moreover, one has to take into account that while case fatality rates are much lower for the younger, they are represent a larger share of the population and their life expectancy is also higher (e.g. over 20 years for the 60 years old). These two factors tend to give more weight to the younger age brackets, leading to resulting parameter estimates similar to ours[41].

One of our main goals has been the introduction of a generic framework, which can be updated by further advances in the accuracy of estimates while still presenting specific results with the data available at this time.

Relation to further studies

A range of determining factors have been examined for the ongoing COVID-19 epidemic, in particular the effect of quarantine[42] and that community-level social distancing may be more important than the social distancing of individuals[43]. An agent-based model for Australia found, in this regard, that school closures may not be decisive[44]. Microsimulation models suggest, on the other hand, that a substantial range of non-pharmaceutical interventions might be needed for an effective containment of the COVID-19 outbreak[6].

We also note attempts to derive disease transmission rates from economic principles of behavior[26], which would allow to measure the cost of the Corona pandemic under different policy settings. Another strand of the literature takes the pandemic as given, and as the basis for scenarios for the economic impact and for the financial-

market volatility[45,46].

---

* Correspondence to gros@itp.uni-frankfurt.de

[1] Sarah Boseley. New data, new policy: why UK's coronavirus strategy changed. *The Guardian*, 2020.

[2] Maegan Vazquez. How Trump's rhetoric has changed as coronavirus spread. *CNN*, 2020.

[3] Koen Deforche, Jurgen Vercauteren, Viktor Müller, and Anne-Mieke Vandamme. Behavioral changes before lockdown, and decreased retail and recreation mobility during lockdown, contributed most to the successful control of the covid-19 epidemic in 35 western countries. *medRxiv*, 2020.

[4] Emeline Han, Melisa Mei Jin Tan, Eva Turk, Devi Sridhar, Gabriel M Leung, Kenji Shibuya, Nima Asgari, Juhwan Oh, Alberto L García-Basteiro, Johanna Hanefeld, et al. Lessons learnt from easing covid-19 restrictions: an analysis of countries and regions in asia pacific and europe. *The Lancet*, 2020.

[5] Annelies Wilder-Smith, Calvin J Chiew, and Vernon J Lee. Can we contain the covid-19 outbreak with the same measures as for sars? *The Lancet Infectious Diseases*, 20(5): e102–e107, 2020. doi:10.1016/S1473-3099(20)30129-8.

[6] Neil M Ferguson, Daniel Laydon, Gemma Nedjati-Gilani, Natsuko Imai, Kylie Ainslie, Marc Baguelin, Sangeeta Bhatia, Adhiratha Boonyasiri, Zulma Cucunubá, Gina Cuomo-Dannenburg, et al. Impact of non-pharmaceutical interventions (npis) to reduce covid-19 mortality and healthcare demand. *Imperial College, London*, 2020. doi: 10.25561/77482.

[7] Susanna Every-Palmer, Matthew Jenkins, Philip Gendall, Janet Hoek, Ben Beaglehole, Caroline Bell, Jonathan Williman, Charlene Rapsey, and James Stanley. Psychological distress, anxiety, family violence, suicidality, and wellbeing in new zealand during the covid-19 lockdown: A cross-sectional study. *PLoS one*, 15(11):e0241658, 2020.

[8] Benjamin M Althouse, Theodore C Bergstrom, and Carl T Bergstrom. A public choice framework for controlling transmissible and evolving diseases. *Proceedings of the National Academy of Sciences*, 107:1696–1701, 2010. doi: 10.1073/pnas.0906078107a.

[9] RR Roberts, EK Mensah, and RA Weinstein. A guide to interpreting economic studies in infectious diseases. *Clinical microbiology and infection*, 16(12):1713–1720, 2010. doi:10.1111/j.1469-0691.2010.03366.x.

[10] Timothy W Russell, Joel Hellewell, Christopher I Jarvis, Kevin van Zandvoort, Sam Abbott, Ruwan Ratnayake, Stefan Flasche, Rosalind M Eggo, Adam J Kucharski, CMMID nCov working group, et al. Estimating the infection and case fatality ratio for covid-19 using age-adjusted data from the outbreak on the diamond princess cruise ship. *medRxiv*, 2020. doi:10.2807/1560-7917.ES.2020.25.12.2000256.

[11] Didier Raoult, Alimuddin Zumla, Franco Locatelli, Giuseppe Ippolito, and Guido Kroemer. Coronavirus infections: Epidemiological, clinical and immunological features and hypotheses. *Cell Stress*, 4(4):66–75, 2020. doi: 10.15698/cst2020.04.216.

[12] Stephanie Bialek et al. Severe outcomes among patients with coronavirus disease 2019 (covid-19)-united states, february 12–march 16, 2020. *MMWR Morb Mortal Wkly Rep*, 69(12):343–346, 2020. doi: 10.15585/mmwr.mm6912e2.

[13] Susan J Tzotzos, Bernhard Fischer, Hendrik Fischer, and Markus Zeitlinger. Incidence of ards and outcomes in hospitalized patients with covid-19: a global literature survey. *Critical Care*, 24(1):1–4, 2020.

[14] CDC. Disease Burden of Influenza. *Center for Disease Control*, 2020.

[15] Jiangzhuo Chen, Anil Vullikanti, Stefan Hoops, Henning Mortveit, Bryan Lewis, Srinivasan Venkatramanan, Wen You, Stephen Eubank, Madhav Marathe, Chris Barrett, et al. Medical costs of keeping the us economy open during covid-19. *Scientific reports*, 10(1):1–10, 2020.

[16] Costs for a hospital stay for covid-19, 2020. URL `https://www.fairhealth.org/article/costs-for-a-hospital-stay-for-covid-19`.

[17] Jörg Martin, Christian Neurohr, Michael Bauer, Manfred Weiß, and Alexander Schleppers. Kosten der intensivmedizinischen versorgung in einem deutschen krankenhaus. *Der Anaesthesist*, 57(5):505–512, 2008. doi:10.1007/s00101-008-1353-7.

[18] Linda Thunstrom, Stephen Newbold, David Finnoff, Madison Ashworth, and Jason F Shogren. The benefits and costs of flattening the curve for covid-19. *SSRN 3561934*, 2020. doi:10.2139/ssrn.3561934.

[19] Andrea Renda, Lorna Schrefler, Giacomo Luchetta, and Roberto Zavatta. Assessing the costs and benefits of regulation. *Brüssel: Centre for European Policy Studies*, 2013.

[20] Sandra Hoffmann. Cost Estimates of Foodborne Illnesses. *United States Department of Agriculture*, 2014.

[21] Peter J Neumann, Joshua T Cohen, Milton C Weinstein, et al. Updating cost-effectiveness the curious resilience of the 50,000-per-qaly threshold. *New England Journal of Medicine*, 371(9):796–797, 2014. doi: 10.1056/NEJMp1405158.

[22] Matthew Rae Twitter et al. Potential costs of coronavirus treatment for people with employer coverage. *Health System Tracker*, 2020.

[23] David M Cutler and Elizabeth Richardson. Your money and your life: The value of health and what affects it. Working Paper 6895, National Bureau of Economic Research, January 1999. URL `http://www.nber.org/papers/w6895`.

[24] Joacim Rocklöv, Henrik Sjödin, and Annelies Wilder-Smith. Covid-19 outbreak on the diamond princess cruise ship: estimating the epidemic potential and effectiveness of public health countermeasures. *Journal of Travel Medicine*, 27, 2020. doi:10.1093/jtm/taaa030.

[25] Matej Mikulic. Health expenditure as a percentage of gross domestic product in selected countries in 2018. *Statista*, 2019.

[26] Martin Eichenbaum, Sérgio Rebelo, and Mathias Trabandt. The macroeconomics of epidemics. *NBER Working Paper No. 26882*, 2020. doi:10.3386/w26882.

[27] Helge Berger, Kenneth Kang, and Changyong Rhee. Blunting the Impact and Hard Choices: Early Lessons from China. *International Monetary Fund*, 2020.

[28] IMF. World Economic Outlook: The Great Lockdown.

International Monetary Fund, 2020.

29 Fernando E Alvarez, David Argente, and Francesco Lippi. A simple planning problem for covid-19 lockdown. Technical report, National Bureau of Economic Research, 2020.

30 Robert Rowthorn and Jan Maciejowski. A cost–benefit analysis of the covid-19 disease. *Oxford Review of Economic Policy*, 36(Supplement_1):S38–S55, 2020.

31 C Jason Wang, Chun Y Ng, and Robert H Brook. Response to covid-19 in taiwan: big data analytics, new technology, and proactive testing. *Jama*, 323(14):1341–1342, 2020.

32 Young Joon Park, Young June Choe, Ok Park, Shin Young Park, Young-Man Kim, Jieun Kim, Sanghui Kweon, Yeonhee Woo, Jin Gwack, Seong Sun Kim, et al. Contact tracing during coronavirus disease outbreak, south korea, 2020. *Emerging infectious diseases*, 26(10):2465–2468, 2020.

33 WHO-China Joint Mission. Report of the WHO-China Joint Mission on Coronavirus Disease 2019 (COVID-19). Geneva 2020, 2020.

34 Ying Liu, Albert A Gayle, Annelies Wilder-Smith, and Joacim Rocklöv. The reproductive number of covid-19 is higher compared to sars coronavirus. *Journal of travel medicine*, 27(2), 2020. doi:10.1093/jtm/taaa021.

35 Hiroshi Nishiura, Tetsuro Kobayashi, Yichi Yang, and Katsuma et al. Hayashi. The rate of underascertainment of novel coronavirus (2019-ncov) infection: Estimation using japanese passengers data on evacuation flights. *J Clin Med.*, 9(2):419, 2020. doi:10.3390/jcm9020419.

36 Jonathan M Read, Jessica RE Bridgen, Derek AT Cummings, Antonia Ho, and Chris P Jewell. Novel coronavirus 2019-ncov: early estimation of epidemiological parameters and epidemic predictions. *MedRxiv*, 2020. doi:10.1101/2020.01.23.20018549.

37 Elisalex Henckel. Coronavirus. *Die Welt*, 2020.

38 Carlo Signorelli and Anna Odone. Age-specific covid-19 case-fatality rate: no evidence of changes over time. *International Journal of Public Health*, 65(8):1435–1436, 2020.

39 Xiang Gao and Qunfeng Dong. A logistic model for age-specific covid-19 case-fatality rates. *JAMIA open*, 3(2): 151–153, 2020.

40 Christian Hoffmann and Eva Wolf. Older age groups and country-specific case fatality rates of covid-19 in europe, usa and canada. *Infection*, pages 1–6, 2020.

41 Daniel Gros. The great lockdown: was it worth it? *CEPS Policy Insights, May, online: https://www. ceps. eu/wp-content/uploads/2020/05/PI2020-11_DG_The-great-lockdown. pdf*, 2020.

42 Liangrong Peng, Wuyue Yang, Dongyan Zhang, Changjing Zhuge, and Liu Hong. Epidemic analysis of covid-19 in china by dynamical modeling. *arXiv:2002.06563*, 2020.

43 Alexander F Siegenfeld and Yaneer Bar-Yam. Eliminating covid-19: A community-based analysis. *arXiv:2003.10086*, 2020.

44 Sheryl L Chang, Nathan Harding, Cameron Zachreson, Oliver M Cliff, and Mikhail Prokopenko. Modelling transmission and control of the covid-19 pandemic in australia. *arXiv:2003.10218*, 2020.

45 Andrew Atkeson. What will be the economic impact of covid-19 in the us? rough estimates of disease scenarios. *NBER Working Paper No. 26867*, 2020. doi: 10.3386/w26867.

46 Richard Baldwin and Beatrice Weder di Mauro. Economics in the time of covid-19, 2020. URL `https://voxeu.org/debates/economics-time-covid-19`.
